# Immune Responses Induced at One Hour Post Cataract Surgery Wounding of the Chick Lens

**DOI:** 10.3390/biom13111615

**Published:** 2023-11-04

**Authors:** JodiRae DeDreu, Morgan D. Basta, Janice L. Walker, A. Sue Menko

**Affiliations:** 1 Department of Pathology and Genomic Medicine, Sidney Kimmel Medical College, Thomas Jefferson University, Philadelphia, PA 19107, USAmorgan.basta@student.jefferson.edu (M.D.B.); janice.walker@jefferson.edu (J.L.W.); 2 Department of Ophthalmology, Sidney Kimmel Medical College, Thomas Jefferson University, Philadelphia, PA 19107, USA

**Keywords:** immune, lens, resident immune cell, injury, wounding, immune response

## Abstract

While the lens is an avascular tissue with an immune-privileged status, studies have now revealed that there are immune responses specifically linked to the lens. The response to lens injury, such as following cataract surgery, has been shown to involve the activation of the resident immune cell population of the lens and the induction of immunomodulatory factors by the wounded epithelium. However, there has been limited investigation into the immediate response of the lens to wounding, particularly those induced factors that are intrinsic to the lens and its associated resident immune cells. Using an established chick embryo ex vivo cataract surgery model has made it possible to determine the early immune responses of this tissue to injury, including its resident immune cells, through a transcriptome analysis. RNA-seq studies were performed to determine the gene expression profile at 1 h post wounding compared to time 0. The results provided evidence that, as occurs in other tissues, the resident immune cells of the lens rapidly acquired a molecular signature consistent with their activation. These studies also identified the expression of many inflammatory factors by the injured lens that are associated with both the induction and regulation of the immune response.

## 1. Introduction

The lens is an avascular tissue with an immune-privileged status [1,2,3], which has resulted in a paucity of studies regarding its immune response when pathogenic insults occur in other regions of the eye or to the wounding of this tissue during cataract surgery. However, it has become clear that, despite its avascularity, there are immune responses specifically involving and targeting the lens [4,5,6,7,8,9,10]. One of the earliest findings of immune cells becoming associated with the lens was in the context of lens dysgenesis in an N-cadherin lens-specific knockout that results in cataract-like lens opacities [4]. In studies with this mouse knockout model, it was discovered that a diverse population of immune cells, including macrophages, T cells, and B cells, are recruited to the lens from its surrounding microenvironment [4]. This finding initiated further investigations into how these innate and adaptive immune responses are recruited to a dysgenic or injured lens.

It is well known that most tissues, including other sites of immune privilege, harbor a population of resident immune cells [11,12,13,14], which typically exhibit properties of dendritic Antigen Presenting Cells (APCs) [15,16,17]. Tissue resident immune cells, which can be sourced from either the yolk sac or the bone marrow, first become positioned within their host tissues during development [18]. This immune cell population adapts to its environment, where it can be maintained into adulthood even without replenishment from the bone marrow [15,16]. Known as the sentinels of danger and injury, resident immune cells are among the first responders to tissue wounding or damage [19,20,21], and have been linked to the activation of both innate and adaptive immune responses [22]. Indeed, like other tissues, the lens becomes populated by resident immune cells during its development [5,23]. It was shown that resident immune cells are a property of lenses across species and that they are maintained into adulthood [5]. A primary source of the lens resident immune cells is the vasculature-rich ciliary body [23]. They access the avascular lens by migrating across the ciliary zonules, fibrils with a fibrillin backbone that extend from the ciliary body to the lens equator where these immune cells migrate across the extracellular matrix-rich lens capsule [5,23]. The lens resident immune cells establish niches amongst the cells of the lens equatorial epithelium, interdigitating with and extending MHCII-rich dendritic process around their neighboring lens epithelial cells [5].

In studies with our established chick embryo ex vivo cataract surgery model we found that the lens resident immune cells are rapidly induced to emerge from their niches among the lens epithelial cells and migrate to the wound site created by the removal of the lens fiber cell mass [5]. In this model, the lens is removed from the eye prior to the surgical injury and the wound response of the lens resident immune cells is followed in explant culture. Immunolocalization studies performed at 1 h post cataract surgery revealed that, in addition to their expression of common immune cell molecules like CD45 and CD18/β2 integrin, the resident immune cells at the wound edge express MHCII and the TLR4 co-receptor CD14, both consistent with an activated APC phenotype [5]. 

Common to both wounded epithelia and their resident immune cells is the upregulation and secretion of cytokines [24,25,26], pivotal factors in the regulation of a tissue’s immune response. Consistent with this canonical wound response, an RNA-seq study performed at 24 h post mouse cataract surgery found there is a significant increase in pro-inflammatory cytokine expression compared to wounding of the contralateral lens just prior to sacrifice [9]. The cytokines induced are consistent with a role in the activation of an innate immune response, with cytokine expression preceding the detection of neutrophils with the post-cataract surgery lens tissue [9]. The upregulation of pro-inflammatory cytokines was also detected in studies performed at 6 h post mouse cataract surgery [27].

Many factors, including cytokines, contribute to the activation of tissue resident immune cells [28], a phenotype characterized by changes in cell morphology and a molecular signature that can include the expression and/or upregulation of molecules like CD83, CD86, CD80, and MHCII [22,29,30,31]. Since the resident immune cells of the lens are rapidly activated to populate the wound edge in response to a cataract-surgery-induced injury to the lens epithelium [5], we now performed an RNA-seq analysis to compare the gene expression profile at 1 h post wounding to that at time 0. As this study was performed using our established chick embryo ex vivo cataract surgery model [32], this RNA-seq analysis allowed us to focus specifically on the injured lens epithelium and its associated resident immune cell population in the absence of any contribution from other regions of the eye. The results revealed that the lens resident immune cells rapidly acquired an “activated” molecular signature and identified inflammatory factors whose expression may play important roles in both resident immune cell activation and in the induction of an immune response to lens injury.

## 2. Materials and Methods

**Figure 1:**
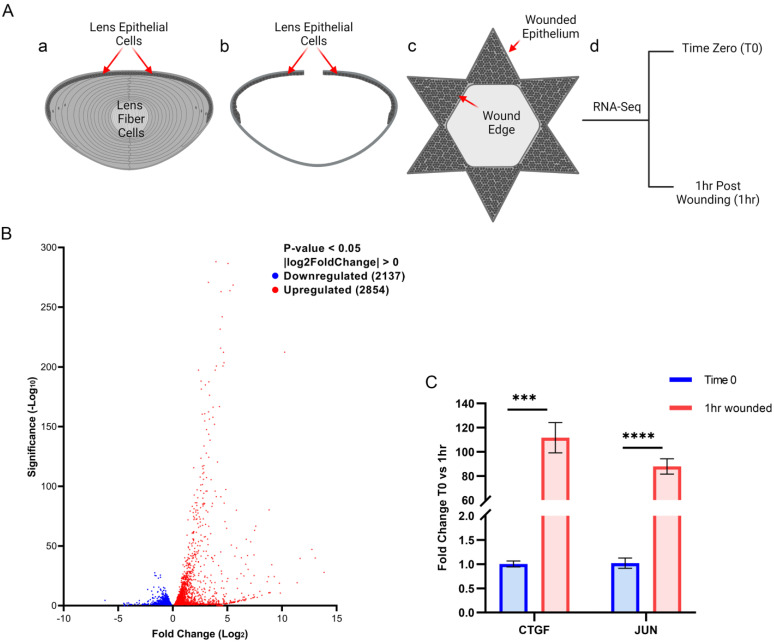
RNA-Seq analysis of ex vivo cataract surgery wounded lenses at 1 h post wounding vs. time zero. (**A**) Diagram of cataract surgery wounding of E14 chick lenses; (**Aa**) model of the chick embryo lens, including the lens epithelium, the lens fiber cells, and the capsule surrounding the lens; (**Ab**) model of the lens after ex vivo cataract surgery wounding removes the fiber cells mass; the wounded lens epithelium and its associated resident immune cells remain linked to the matrix capsule surrounding the lens; (**Ac**) model of wounded lens explants cultured for 1 h post cataract surgery; (**Ad**) explants were collected for RNA-Seq analysis at time zero (T0) and at 1 h post wounding. (**B**) A volcano plot showing the distribution of downregulated (blue) and upregulated (red) genes at 1 h post wounding compared to T0 with a *p*-value < 0.05 and log2FoldChange > 0, plotted as Log2fold change vs Significance (−Log10). (**C**) Increased expression of CTGF and JUN at 1 h post injury as determined by qRT-PCR analysis. Error bars represent S.E.M., *** *p* ≤ 0.001, **** *p* ≤ 0.00001.

Ex vivo cataract surgery chick model. Whole lenses are removed from embryonic chicks at day 14 (Figure 1A(a)). An opening is made in the anterior region of the lens by removing the central lens epithelium. The lens fiber cells, which are weakly attached to their underlying basement membrane capsule, are removed via hydroelution. This approach leaves behind the lens capsule, its attached lens equatorial epithelial cells, and associated lens resident immune cells (Figure 1A(b)) [5]. These post-cataract surgery explants were examined by RNAseq either at the time of wounding (time 0), or at 1 h post-wounding. For the 1 h time point, the wounded lens explant was flattened, capsule side down, on a tissue culture substrate after making incisions into the lens equatorial epithelium. In this star-shaped explant, the wound edge is located at the site where the equatorial epithelium meets the region of the posterior lens capsule from which the fiber cells were removed (Figure 1A(c)). The wounded lens explants were cultured for 1 h in complete media (M199 (Gibco 11150-059) containing 10% FBS (Gibco A31605-01), 1% L-Glut (Corning 25-005-CI), and 1% Pen-Strep (Corning 30-002-CI)) at 37 °C with 5% CO_2_. 

RNA-Seq and Bioinformatics Analysis. For this study, 6 tissue explants were collected at each of the two time points, directly after wounding (time zero (T0)), and at 1 h post wounding. Three individual experimental samples of 6 explants per time point were sent to Novogene for RNA sequencing using an Illumina platform. Novogene provided RNAseq results and analysis including differential gene expression (DEGs), log2fold change, pvalue, adjusted pvalue (padj), and bioinformatics analysis, including gene ontology (GO).

qRT-PCR Total RNA was isolated at time 0 after mock cataract surgery and 1 h post wounding from the ex vivo post-cataract-surgery cultures using Qiagen RNeasy Mini Kit (74004, Qiagen, Germantown, MD, USA). RT-qPCR was performed on a QuantStudio™ 5 System using 2X Power SYBER green PCR master mix (4309155, Thermo Fisher, Waltham, MA, USA). PCR analyses were conducted in triplicate or quadruplicate for each sample. The sequences for the primers include the following genes in chicken (gallus gallus): *CTGF* forward primer 5′ AAGACACTTACGGCCCAGAC 3′, and reverse primer 5′ TTGGAGCAAGCACTCCACTC 3′, *Jun* forward primer 5′ AACTCAGAGCTGGCATCCAC 3′, and reverse primer 5′ TTAGCATTAGCTGGCACCCG 3′, and *GAPDH* forward 5′ ATTTGGCCGTATTGGCCGCC 3′, and reverse 5′ AGTGCCCTTGAAGTGTCCGTGT 3′. Gene expression changes were calculated using the 2-∆∆CT method and *GAPDH* was used as an internal control.

DEGs, GO Pathway Enrichment Analysis, and Statistical Analysis. Adjusted p-values (padj) based on the Benjamini and Hochberg method were provided by Novogene. Genes were considered differentially expressed (DEGs) based on their calculated padj. A volcano plot was created from the DEGs (both upregulated and downregulated) based on the padj values provided by Novogene after determining their significance (−Log10) using Excel. GO bioinformatic analysis was provided by Novogene, and the significance (−Log10) of these pathways was calculated from their padj using Excel prior to graphing the results. Analysis of individual molecules upregulated at 1 h post wounding was also determined by calculating the significance (−Log10) from the padj prior to graphing. For upregulated molecules that are linked to the activation of resident immune cells, the fold increase was graphed using the log2fold increase data provided in the bioinformatics analysis from Novogene. Prism version 9 was used to create the graphs presented in these studies. Cytoscape_v3.9.1 software was used to determine the protein–protein interactions (PPI) between different groups of DEGs of interest with a *p*-value < 0.05 [33]. 

Immunofluorescence labeling. Ex vivo wounded lens explants were cultured for 1 h or 4 h and fixed in 4% para-formaldehyde for 15 min before immunolabeling. Samples were permeabilized (0.25% TritonX100 in PBS) for 30 min at room temperature, blocked with 5% goat or donkey serum in PBS for 30 min at room temperature, incubated in primary antibody diluted in 0.1% Tween20 in PBS for 3 h 37 °C, washed with PBS, and then incubated in secondary antibody diluted in 0.1% Tween20 in PBS for 30 min at 37 °C. Samples were washed with PBS before mounting with ProLong Diamond Antifade Mountant (Invitrogen P36970). The primary antibodies used for these studies included S100A4 (DSHB CPTC-S100A4-1; dilution 1:10), CD44 (DSHB 1D10; dilution 1:10), and Beta2 integrin (SantaCruz sc-6623; dilution 1:100).

## 3. Results

### 3.1. RNA-Seq Analysis Reveals That Lens Wounding Rapidly Induces an Extensive Immune Response

The immediate response of lens resident immune cells to wounding, and of the epithelium in which they reside, was examined in this study with our established chick embryo ex vivo cataract surgery model (Figure 1A). To create this clinically relevant injury model, the lens is removed from the eye before cataract surgery is performed ex vivo using an established procedure that involves extraction of the differentiated fiber cell mass [34]. The wounded lens epithelium, together with its associated, injury-activated resident immune cells, remain linked to the basement membrane capsule that surrounds the lens [35,36]. The immediate immune responses to this injury were investigated at the transcriptome level using RNA-seq analysis, comparing the transcripts induced at 1 h after placing the wounded explants in culture to those expressed at the time of wounding (T0) (Figure 1). The volcano plot in Figure 1B shows that of the 4991 differentially expressed genes (DEGs), 2137 were downregulated (blue) and 2854 were upregulated (red) at 1 h post wounding.

The top 50 upregulated DEGs are listed in order of their significance (−Log10) (Table 1; with original data provided in Supplemental File S1). They included *CTGF*, a matricellular protein involved in wound repair [37], and the transcription factors *IRF8*, *JUN*, and *STAT3* that regulate myeloid cell differentiation, cytokine production, and cytokine signaling, respectively [38,39,40]. *JUN* is a classic immediate early gene responsive to many cellular stimuli. qRT-PCR performed at the time of wounding and at 1 h post wounding for *CTGF* and *JUN* showed that these early response genes were rapidly upregulated in response to lens wounding (Figure 1C), confirming the RNA-seq results (Table 1).

**Figure 2:**
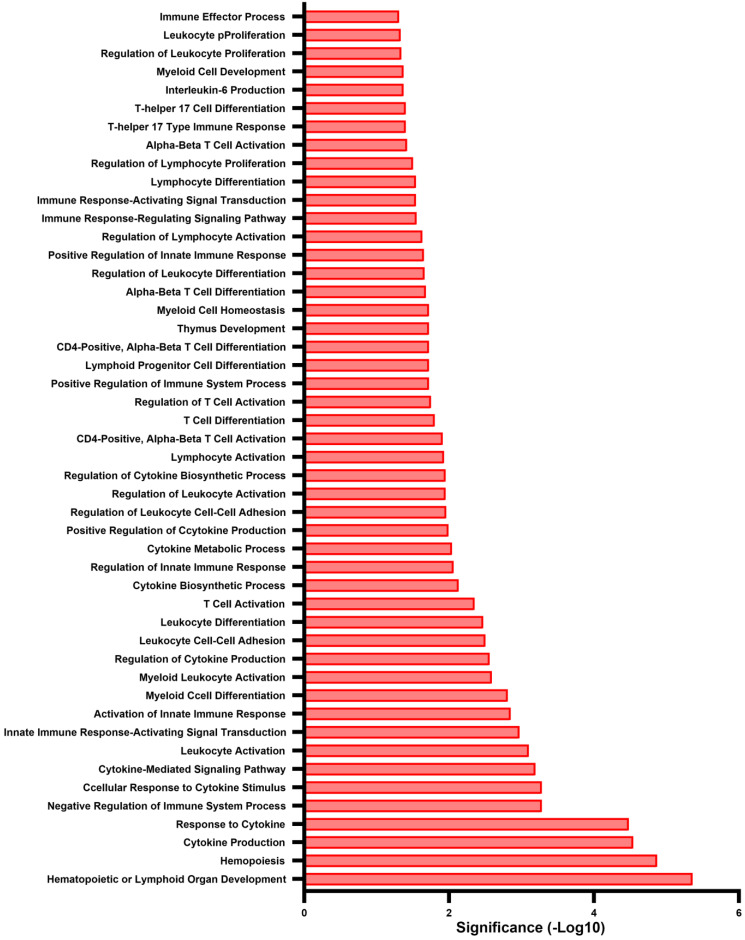
GO pathways induced at 1 h post lens wounding. Forty-eight immune-linked gene ontology (GO) biological processes induced at 1 h post lens wounding, graphed based on their significance (−Log10) values.

**Figure 3:**
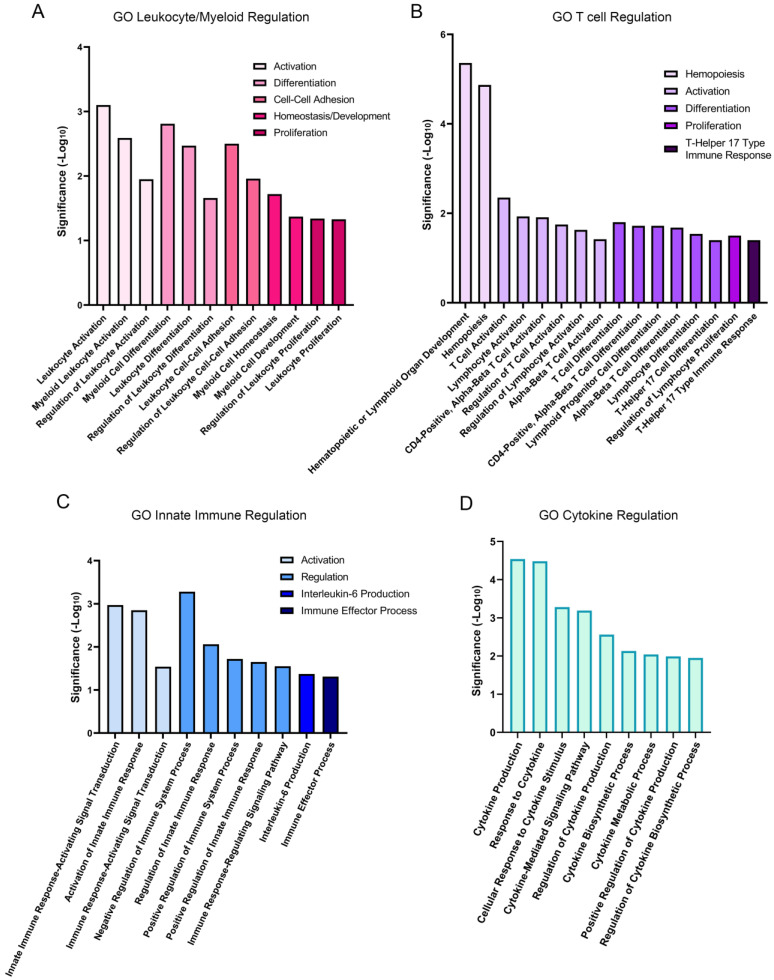
Immune-pathway-linked gene ontology groups. Immune-linked GO processes are presented in four distinct groupings, including (**A**) leukocyte/myeloid regulation, (**B**) T cell regulation, (**C**) innate immune regulation, and (**D**) cytokine regulation, and graphed based on significance (-Log10) of the functional subgroups.

**Figure 4:**
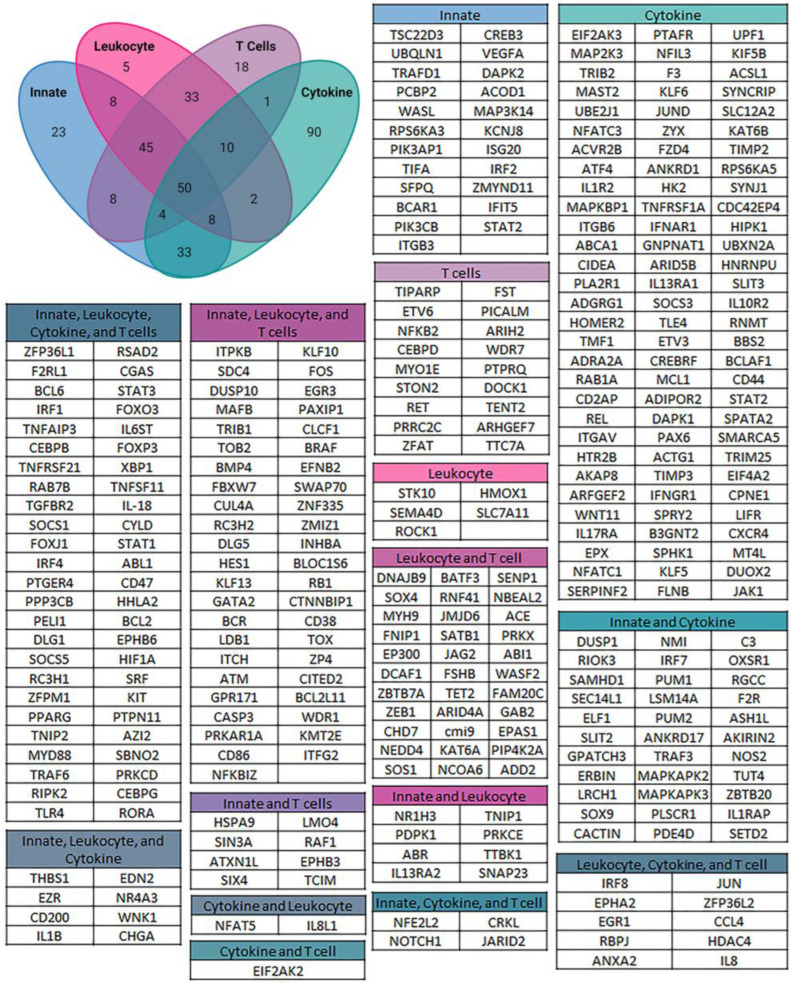
Unique and shared genes between the 4 immune GO groups. The Venn diagram illustrates the number of upregulated genes that are unique and shared between each group, with a total of 338 genes identified. These genes were then listed in different groups according to the GO groups they are associated with and color coded to match the Venn diagram. For each of the 4 immune pathway GO groups, there were unique genes, 23 associated with the innate immune regulation group, 5 associated with the leukocyte/myeloid regulation group, 18 associated with the T cell regulation group, and 90 associated with the cytokine regulation group. Various number of genes were shared between each of these four groups, with 50 genes associated with all four groups.

Gene ontology (GO) analysis revealed the biological processes that were significantly enriched at 1 h post wounding. Of a total of 877 upregulated GO processes that were identified, 48 were linked to the immune response (Figure 2). These pathways fell into four separate groups that highlight the extensive impact of lens wounding on induction of an immune response (Figure 3). They include pathways involved in leukocyte/myeloid regulation (Figure 3A), T-cell induction/regulation (Figure 3B), the innate immune response (Figure 3C), and induction/regulation of cytokines (Figure 3D). Figure 4 shows the specific genes induced at 1 h post lens wounding that were linked to leukocyte, T cell, cytokine, or immune response pathways, and those induced genes that function in two or more of these pathways. For example, induction of the messenger RNA for Janus kinase 1 (*JAK1*) is uniquely associated with cytokine signaling [41].

### 3.2. Resident Immune Cells of the Lens Are Rapidly Activated in Response to Wounding

**Figure 5:**
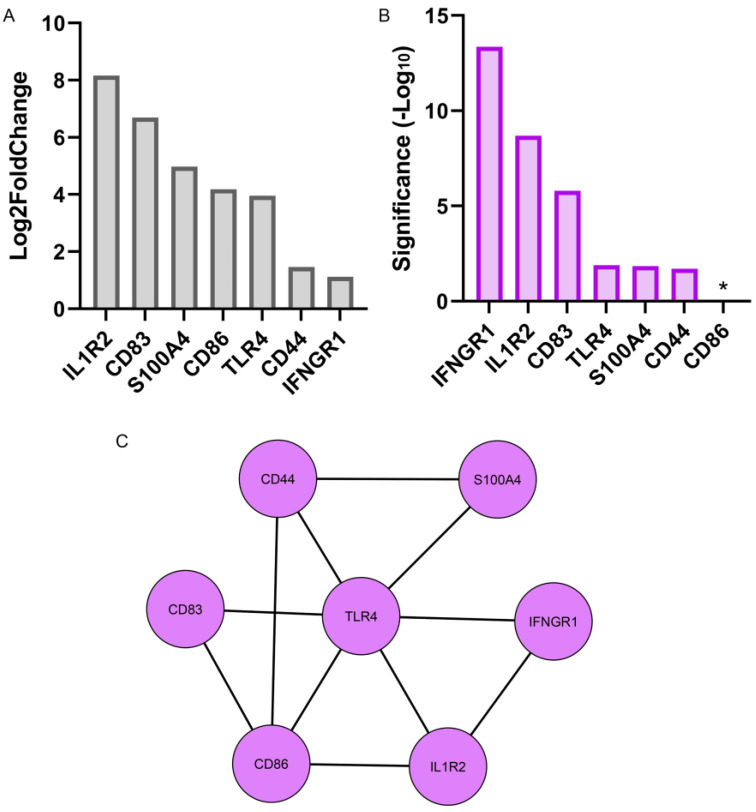
Molecular signature of activated resident immune cells at 1 h post lens wounding. (**A**) Graphical representation of the log2fold change and (**B**) significance (−Log10) for 7 genes induced at 1 h post wounding associated with the activation of resident immune cells including *CD83, CD86, TLR4*, *CD44*, *S100A4*, *IL1R2*, and *IFNGR1*. * *p*-value = 0.03; (**C**) Cytoscape analysis showing the interactome of these 7 molecules.

Resident immune cells are known as a tissue’s immediate responders to injury, often a dendritic immune cell type that functions as Antigen Presenting Cells (APCs) [15,16,17]. We previously discovered that the lens, despite its property of being an avascular tissue, becomes populated by resident immune cells during development that comprises about 3% of the total cell population of this tissue [5,42]. The lens resident immune cells exhibit a dendritic morphology, and are interdigitated among the cells of the lens epithelium [5]. The RNA-seq revealed that there was a Log2fold increase in *CD83* and CD86 at 1 h post-lens wounding (Figure 5A). Both of these molecules are considered to belong to the molecular signature of activated APCs [43], thereby providing direct evidence that the resident immune cell subpopulation of the lens was rapidly activated in response to cataract surgery injury. Other genes characteristic of an activated immune cell phenotype that were induced at 1 h post injury included *CD44* (a cell adhesion molecule whose functions include immune cell migration and antigen presentation) [44], *TLR4* (Toll-like receptor 4, whose activation can lead to the production of inflammatory cytokines) [45], *S100A4* (which mediates macrophage recruitment and chemotaxis) [46], *IL-1R2* (a decoy receptor for IL-1 that is expressed by immune cells) [47], and *IFNGR1* (a component of IFNGR expressed by macrophages and whose ligand binding induces JAK/STAT signaling) [48] (Figure 5A). The significance of the increase in expression of each of these immune cell molecules is shown based on their significance (−Log10), which was calculated from their adjusted *p* values (Figure 5B). The complex network of interactions between the molecules induced in lens resident immune cells in response to wounding are highlighted in the interactome shown in Figure 4C, created using Cytoscape software [33]. In addition to these molecules, high levels of the messenger RNA for *CD38*, a surface glycoprotein expressed by immune cells [49,50], were also expressed at 1 h post injury but at similar levels to its expression at T0. 

**Figure 6:**
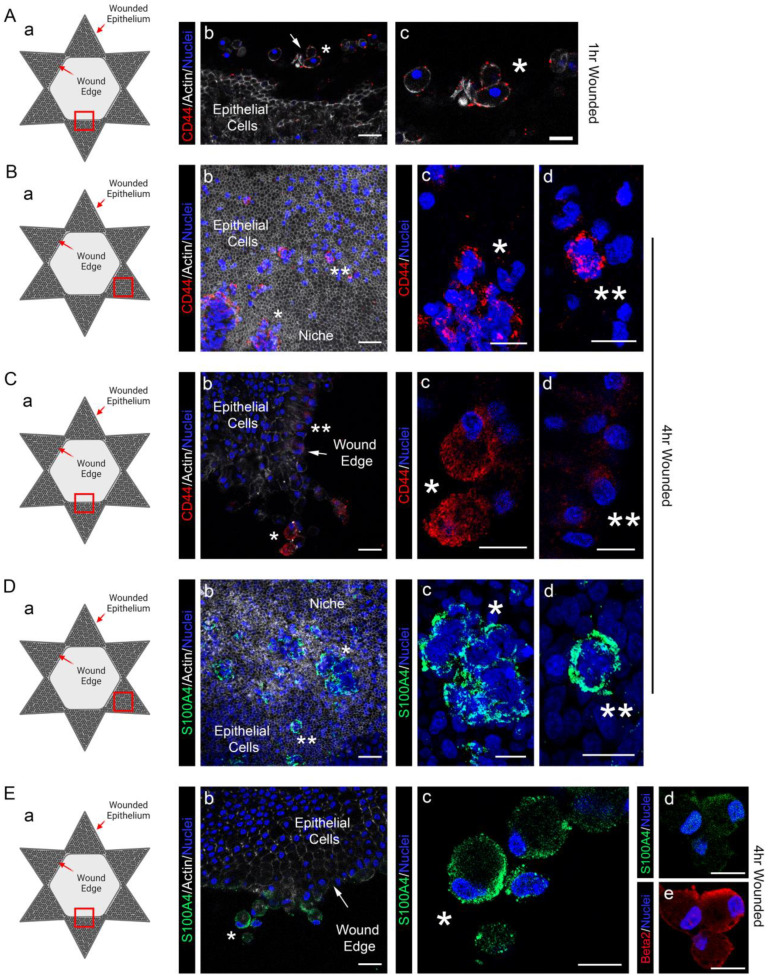
CD44 and S100A4 are specifically localized to the resident immune cells of the lens post cataract surgery wounding. (**A**–**E**) Confocal microscopy images of wounded lens explants at (**A**) 1 h and (**B**–**E**) 4 h post wounding immunolabeled for the resident immune cell proteins (**A**–**C**) CD44 (red) or (**D**,**E**) S100A4 (green), each also labeled for nuclei with DAPI (blue). (**Ab**,**c**,**Bb**,**Cb**,**Db**,**Eb**) were co-labeled for F-actin (white). In (**Ed**,**e**) explants were co-immunolabeled for S100A4 (green) and β2 integrin/CD18 (red). Images were acquired with a Zeiss 800LSM confocal, and 40× Z-stacks collected of resident immune cells at the wound edge (**A**,**C**,**E**, arrows) and of niches of resident immune cells among the lens epithelium (**B**,**D**). (**Aa**,**Ba**,**Ca**,**Da**,**Ea**) diagram the wounded explants illustrate the site at which each image was acquired. (**Ab**,**Bb**,**Cb**,**Db**,**Eb**) are low magnification overviews, mag bar = 20 µm, with asterisks (* and **) denoting the regions from which the higher magnification images in (**Ac**,**Bc**,**d**,**Cc**,**d**,**Dc**,**d**,**Ec**–**e**) were acquired, mag bar = 10 µm. Images in (**Ab**,**c**,**Cb**–**d**,**Db**–**d**,**Eb**–**e**) are single optical planes; (**Bb**–**d**,**Db**–**d**) are projection images.

Our previous studies show that among the characteristics of the resident immune cells of the lens induced to rapidly migrate to the wound edge of the epithelium following ex vivo chick embryo lens cataract surgery specifically express the immune cell molecules CD45, CD18/β2 integrin, the TLR4 co-receptor CD14, and CD44 [5]. CD44 is a cell surface receptor that mediates immune cell migration and also plays a role in antigen presentation [44]. Studies with a mouse model of lens epithelial cell injury also found that CD44 was induced [51]. We now show for the first time that both messenger RNA (Figure 5A) and protein (Figure 6A) for CD44 were induced rapidly, within 1 h post wounding (Figure 6A(a–c)). The immunolocalization analyses also revealed that CD44 was expressed exclusively by the resident immune cells at the wound edge, and not by the wounded lens epithelial cells (Figure 6A–C). Studies performed at 4 h post wounding showed that CD44 was highly expressed by both resident immune cells that had populated the wound edge and by resident immune cells that had remained in their original niches interspersed amongst the cells of the lens epithelium (Figure 6B(a-d),C(a–d)). Both these resident immune cell populations also expressed high levels of S100A4 (Figure 6Da–d,Ea–c), a molecule whose messenger RNA was rapidly induced within one hour of lens wounding (Figure 5A), which functions in macrophage recruitment and chemotaxis [46]. S100A4 was not detected in the cells of the wounded lens epithelium, a result consistent with previous studies showing a lack of S100A4 expression by lens epithelial cells [52]. Co-immunolabeling for S100A4 and CD18/β2 integrin provided additional confirmation that the S100A4+ cells activated in response to lens wounding have an immune cell phenotype (Figure 6E(d,e)).

### 3.3. RNA-Seq Analysis Shows That Transcription Factors Linked to the Immune Response Are Early Responders to Lens Wounding 

**Figure 7:**
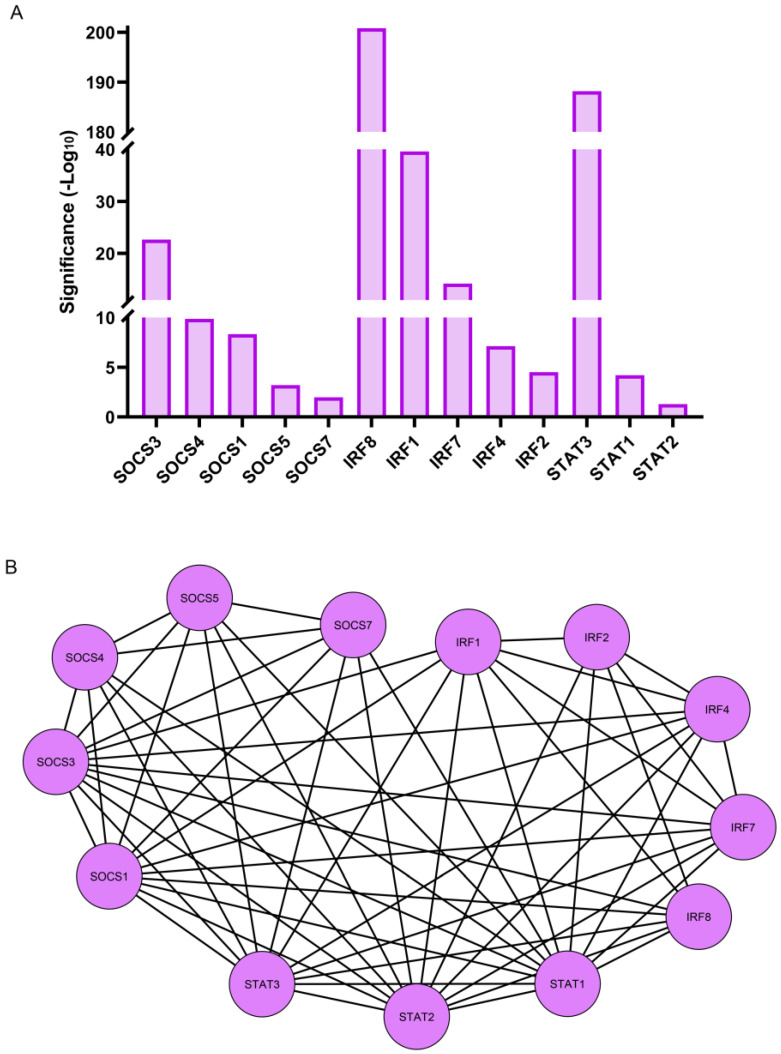
Differentially expressed transcription factor genes induced at 1 h post lens wounding. (**A**) Graphical representation of the transcription factors genes that were significantly upregulated between time 0 and 1 h post lens wounding. These include members of three distinct transcription factor families and included *SOCS1/3/4/5/7*, *IRF1/2/4/7/8*, and *STAT1/2/3*. Significance is based on (−Log10). (**B**) Cytoscape analysis demonstrates the interactome of these 13 transcription factors, with 58 interactions identified.

RNA-seq analysis revealed that three transcription factor families, all key regulators of the immune response, were among those most significantly induced at 1 h post lens wounding. They included multiple members of the SOCS (suppressor of cytokine signaling), IRF (interferon regulatory transcription factor), and STAT (signal transducer and activator of transcription) families of transcriptional regulators (Figure 7A). The induced SOCS transcription factors included *SOCS1*, *SOCS3*, *SOCS4*, *SOCS5*, and *SOCS7*. These factors have known functions in pathways that are involved in the attenuation of cytokine signaling and in the determination of macrophage/dendritic cell fate [53,54]. The IRF family members that were upregulated within 1 h of lens wounding included *IRF1*, *IRF2*, *IRF4*, *IRF7*, and *IRF8*. This transcription factor family is linked to pathways involved in regulating innate and adaptive immune responses such as interferon expression, pro-inflammatory responses, and the differentiation of dendritic, myeloid, and T cells [55,56]. Three members of the STAT family, *STAT1*, *STAT2*, and *STAT3* were also induced within 1 h of lens wounding. This family of transcription factors functions in the JAK signaling pathway that is activated downstream of cytokine receptor binding [57,58]. The interactome of these transcription factors is shown in Figure 7B.

### 3.4. Gene Expression of a Specific Subset of Cytokines and Cytokine Receptors Were Induced within 1 h of Lens Injury

**Figure 8:**
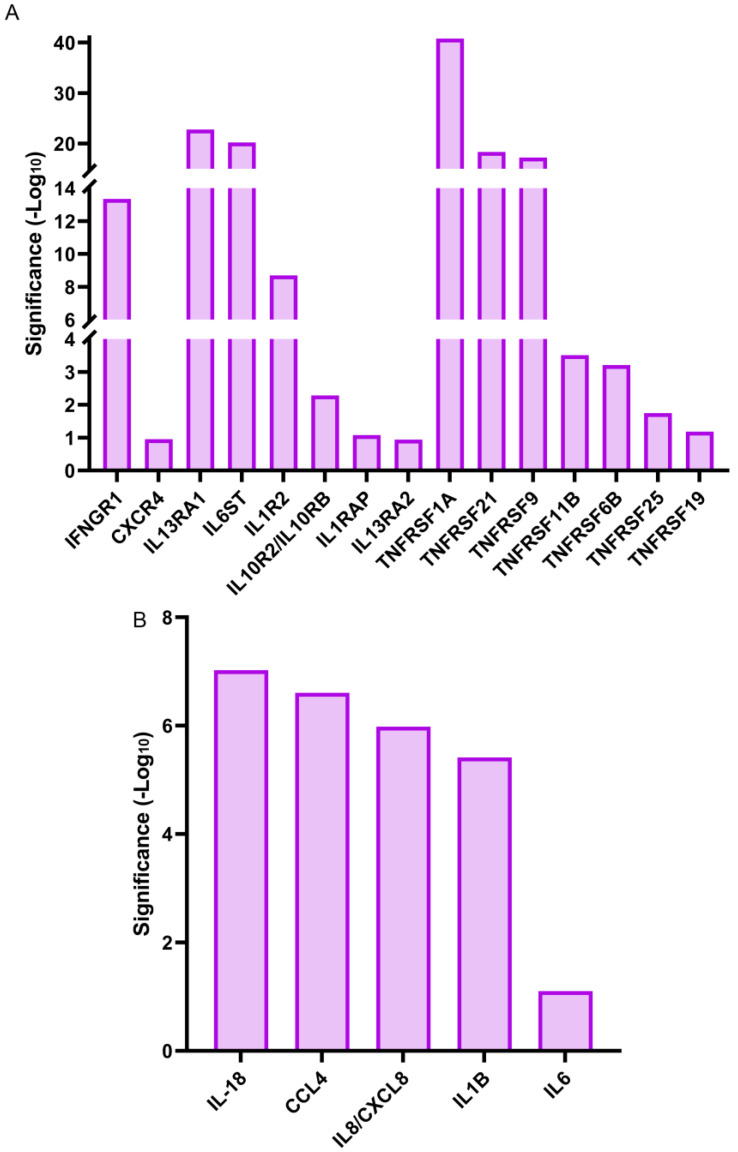
Induction of cytokine receptors and cytokines genes at 1 h post lens wounding. Graphical representation of the (**A**) cytokine receptors and (**B**) cytokines whose gene expression was significantly upregulated between time 0 and 1 h post lens wounding. Gene expression of 15 cytokine receptors were induced including the interleukins (ILs) *IL1R2*, *IL10R2/IL10RB*, *IL13RA1/2*, *IL1RAP*, *IL6ST*, *CXCR4*, *IFNGR1*, and tumor necrosis factors (TNFs) *TNFRSF11B*, *TNFRSF19*, *TNFRSF1A*, *TNFRSF21*, *TNFRSF25*, *TNFRSF6B*, and *TNFRSF9*. Six cytokine genes were induced, including *IL6*, *IL8/CXCL8*, *IL18*, *IL1B*, and *CCL4*. Significance is based on (−Log10).

**Figure 9:**
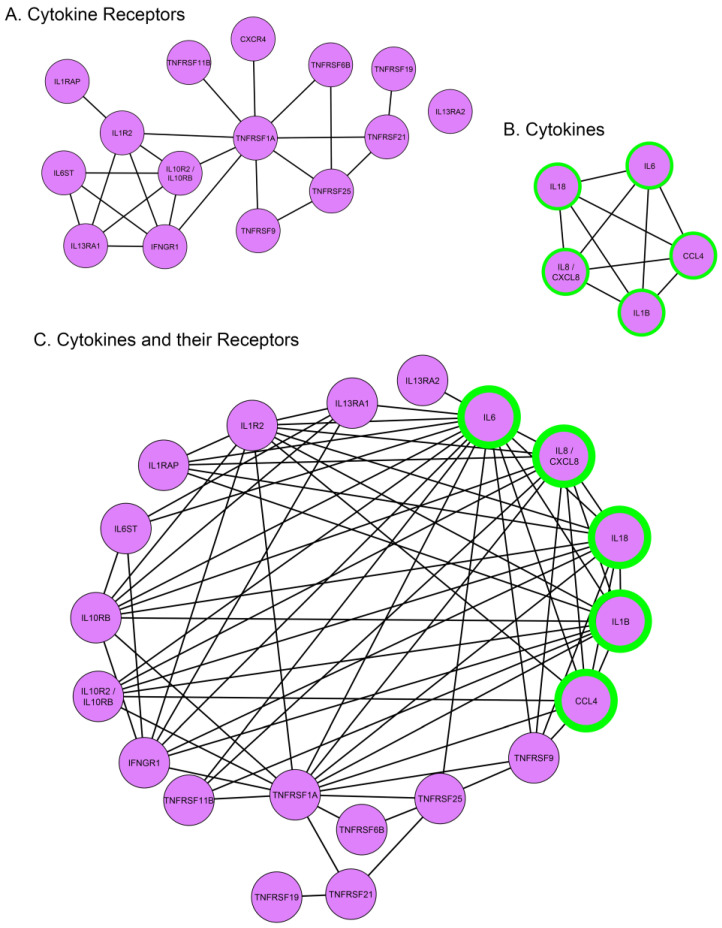
Cytokine/cytokine receptor interactomes at 1 h post lens wounding. The interactions of induced (**A**) cytokine receptors, (**B**) cytokines, and (**C**) cytokines, together with cytokine receptors created with Cytoscape analysis, showed there are 23 interactions among the 14 of the induced cytokine receptors, with none for *IL13RA2*, 10 interactions among the 6 cytokine genes, and 70 interactions when cytokines and cytokine receptors are analyzed together. The Cytoscape analysis for cytokines and cytokine receptors shows an interaction between *IL13RA2* and *IL6*. The cytokine genes are shown with a green edge to easily distinguish them from the cytokine receptors in the Cytoscape analysis shown in (**C**).

Cytokines, like interleukins, play crucial roles in the regulation and induction of the immune response [59]. In response to wounding, they can be expressed either by activated immune cells or by the cells of the injured tissues [60,61]. Our RNA-seq analysis showed that gene expression for a small number of cytokines, mostly interleukins, were significantly upregulated within 1 h of lens wounding (Figure 8B). These included the cytokines *IL6*, *IL18*, and *IL1B*, and the chemokines *IL8/CXCL8*, and *CCL4*. In contrast, there were many cytokine receptors that were significantly upregulated at 1 h post lens wounding, including interleukin receptors like *IL1R2* and the receptor for interferon γ, *IFNGR1*, chemokine receptors like *CXCR4*, and a number of tumor necrosis factor receptors, including *TNFRSF1A* (Figure 8A). The interactomes are shown individually for the cytokines and cytokine receptors (Figure 9A,B) that were significantly induced at 1 h post lens wounding, as well as the interactions between these cytokines and cytokine receptors (Figure 9C, cytokines highlighted with a green outline).

## 4. Discussion

In this study, we reveal the early changes in the transcriptome as related to the lens immune response to injury, comparing time 0 to 1 h post cataract surgery wounding. An important aspect of these studies is that this RNA-seq analysis was performed with lenses on which the cataract surgery wounding was performed ex vivo, after their removal from the eye. This approach made it possible to: (1) reveal changes in the immune response intrinsic to the lens itself in the absence of influence by other eye tissues and (2) identify changes in transcripts that are specifically induced in resident immune cells of the lens following cataract surgery wounding in the absence of contributions from immune cells recruited from regions of the eye outside of this tissue. Overall, our findings establish the lens’s ability to elicit a controlled immune response to insult. We provide evidence of changes in the transcriptome that support an activated molecular signature of the lens resident immune cells, as well as changes in cytokine, cytokine receptor, and transcription factor gene expression that imply the ability of the cells of the injured lens, both resident immune cell populations and the wounded lens epithelium, to modulate the extent of the lens immune response to wounding. 

Comparison of RNA-seq analysis performed on ex vivo wounded lens tissue at 1 h post injury to that at the time of wounding (T0) revealed the induction of a molecular signature of activated Antigen Presenting Cells by the lens resident immune cells. Principal among the differentially expressed genes was *CD83*, a membrane bound member of the immunoglobulin superfamily that is expressed on the surface of activated APCs, including dendritic immune cells [62]. The induction of *CD83* at 1 h post lens wounding provides strong evidence that the lens resident immune cells, immune cells with a dendritic morphology [5], are APCs. Two other molecules associated with immune cells, the cell surface adhesion receptor *CD44* [44] and *S100A4*, a mediator of macrophage recruitment and chemotaxis [46], were also significantly upregulated in response to the wounding of the lens ex vivo. Immunolocalization studies confirmed that these molecules were expressed exclusively by the lens resident immune cells, both by the resident immune cells that had migrated to the wound edge and those that had remained in niches interdigitated among the lens epithelial cells. The function of these wound-activated lens resident immune cells is informed by the induction of two common immune cell receptors, Toll-like receptor 4 (*TLR4*), which plays a role in the production of inflammatory cytokines [45], and interferon γ receptor 1 (*IFNGR1*), a component of the interferon gamma receptor IFNGR expressed by macrophages whose ligand binding induces JAK/STAT signaling [48,63]. These findings reveal unique properties associated with the resident immune cell functional response to lens wounding.

Studies with in vivo mouse models, including mutations that result in lens dysgenesis [4,8,10] and cataract surgery wounding [9], show that immune cells, including macrophages and neutrophils, are recruited to lens dysgenic/wound sites from other regions of the eye. However, they did not address the specific response of the lens-associated resident immune cells, including their role in orchestrating the recruitment of immune cells to the lens and modulating the functional outcome of the lens to insult. Previous RNA-seq analyses showed the induction of immediate early transcription factors, including Egr1 and FosB, in addition to pro-inflammatory cytokines, at 6 h after ex vivo mouse cataract surgery, and that Egr1 and FosB were also induced within 6 h of lens epithelial wounding performed ex vivo [27]. In the mouse cataract surgery model, it was also found that, by 24 h post surgery, genes associated with the innate immune response are induced [9]. Our RNAseq study of genes induced in response to lens wounding was conducted at an earlier time post wounding, just 1 h following chick lens cataract surgery performed ex vivo following isolating the lenses from the eye. This approach made it possible to focus on the response of the injured epithelium and its associated resident immune cells in the absence of any immune contribution from outside of the lens. In this reductionist lens wounding model, we found that the immediate early transcription factors were induced within 1 h of injury, including the transcription factors Egr1 and FosB (Supplemental File S1), which had been previously reported for the mouse model at 6 h post cataract surgery [27]. Also, consistent with studies performed at later times post lens wounding, we found that pro-inflammatory cytokine genes are induced within 1 h post lens wounding, which at this early time included the chemokine *CCL4*, also known as macrophage inflammatory protein. *CCL4* functions as a chemoattractant that attracts immune cells and induces their migration from the vasculature to peripheral tissues [64]. Our findings also showed a significant increase in the expression of many cytokine receptors. These included interleukin receptors, the *CXCR4* chemokine receptor, and multiple members of the tumor necrosis factor receptor superfamily, some of which function as death receptors. The cytokines and cytokine receptors that we found were upregulated in the wounded lens tissue included ones with pro-inflammatory functions and others that function in the regulation of the immune response. These findings reveal a complex regulation of cytokine/cytokine receptor expression at the transcriptome level following lens wounding. An important area for future research will be determining whether these cytokines are produced by the wounded lens epithelial cells and/or the lens resident immune cells, and the cell-specificity of cytokine receptor expression. Such information will be required to decipher the coordinate function of these cells in modulating the outcome of lens injury. 

Our RNAseq findings revealed that members of three distinct transcription factor families with links to the immune response were among the earliest responders to lens wounding. Their expression could be induced in cells of the wounded lens epithelium and/or the activated resident immune cells. The transcription factors induced in response to lens wounding included members of the *SOCS*, *IRF*, and *STAT* families. Interestingly, their known functions include both the induction and suppression of cytokines, as well as roles in the differentiation of immune cells, including of macrophages and dendritic cells. While further studies are needed to determine the specific function of any of these induced transcription factors, it is tempting to speculate that the response to lens wounding includes the ability to induce an immune response, as well as to regulate the extent of that response. For example, both the interferon regulatory transcription factors *IRF1* and *IRF2* are induced within 1 h of lens wounding. *IRF1* is induced by type 1 IFNs to promote inflammation, while *IRF2* is a competitive inhibitor of IRF1, driving an interferon suppressive program [65] and able to inhibit pro-inflammatory responses in macrophages [66]. This type of balancing act would be considered very important in the context of an organ like the eye, where maintaining/restoring homeostasis is key to preserving clear vision.

The results of these RNA-seq studies have provided the first evidence of the early activation of lens resident immune cells to wounding, and support the conclusion that the response of the lens to wounding is one that is carefully regulated, allowing the activation of an immune response while simultaneously providing factors that regulate this response and prevent it from resulting in damage to the eye.

## Figures and Tables

**Table 1:** Top 50 differentially expressed genes (DEGs). The top 50 DEGs, shown in order of their significance (−Log10). For the top 8 induced messenger RNAs * represents a significance (−Log10) with a padj greater than the highest padj reported of 7.67E-289, and listed in the analytics as 0.

DEGs		Significance (-Log10)	DEGs		Significance (-Log10)
1	CTGF	*	26	SNRK	187.5951835
2	JUN	*	27	CBX4	185.0514131
3	BTG2	*	28	BHLHE40	181.4359902
4	GADD45B	*	29	ANGPTL4	176.3661648
5	CISH	*	30	LONRF3	166.8524546
6	NFIL3	*	31	NET1	166.375484
7	MYC	*	32	MSX1	162.4118963
8	CREM	*	33	EIF2AK3	160.8609588
9	F3	288.1154735	34	MAFK	157.9399122
10	KLF6	286.5836756	35	VCL	156.3186263
11	TGIF1	270.8078907	36	EPHA2	154.7870455
12	FOSL2	268.5125469	37	EDN2	152.0153907
13	PCDH9	263.8520327	38	SRF	147.5372354
14	ERRFI1	263.0684018	39	PLK3	143.6585299
15	ARID5A	241.9775771	40	POLE	138.7371147
16	DUSP8	231.57357	41	MAP3K14	133.8617124
17	MAFF	215.6329808	42	IER5	127.8269684
18	ATF3	212.2826148	43	BACH1	124.3708318
19	MAPKKK3L	212.1738763	44	ZFP36L1	123.5899623
20	PIM1	203.6009763	45	SERTAD2	120.4135481
21	LONRF1	200.8374542	46	ITPKB	117.3457473
22	IRF8	200.8362272	47	KLF11	116.8070514
23	BTG1	197.4431125	48	THBS1	115.5955296
24	PIM3	197.4431125	49	PISD	114.842178
25	STAT3	188.1798597	50	SGMS2	111.7124197

## Data Availability

The RNA-sequencing data have been deposited at the Gene Expression Omnibus (GEO) and are publicly available as of the date of publication. The accession number is GSE239368.

## Supplementary Materials

The following supporting information can be downloaded at: https://www.mdpi.com/article/10.3390/biom13111615/s1, Excel file: Onehour vs. T0 deg.
